# Objective Assessment of Syndesmosis Stability Using the Hook Test

**DOI:** 10.3390/jcm12144580

**Published:** 2023-07-10

**Authors:** Jakob Hallbauer, Philipp Schenk, Lea Herrmann, Bernhard Wilhelm Ullrich, Uta Biedermann, Britt Wildemann, Gunther Olaf Hofmann, Felix Christian Kohler

**Affiliations:** 1 Department of Trauma, Hand and Reconstructive Surgery, Jena University Hospital, Friedrich Schiller University Jena, 07747 Jena, Germany; herrmann.wulf@gmx.net (L.H.); britt.wildemann@med.uni-jena.de (B.W.); gunther.hofmann@med.uni-jena.de (G.O.H.); felix.kohler@med.uni-jena.de (F.C.K.); 2 Reseaserch Executive Department, BG Klinikum Bergmannstrost, 06112 Halle, Germany; philipp.schenk@med.uni-jena.de; 3 Department of Trauma and Reconstructive Surgery, BG Klinikum Bergmannstrost, 06112 Halle, Germany; bernhard.ullrich@bergmannstrost.de; 4 Institute of Anatomy I, Jena University Hospital, Friedrich Schiller University Jena, 07743 Jena, Germany; uta.biedermann@med.uni-jena.de

**Keywords:** trauma surgery, syndesmosis, clinical test, upper ankle joint, biomechanics, cadaver

## Abstract

The hook test is a widely used intraoperative method for assessing syndesmosis stability. However, there are no recommendations regarding the force required to perform this test. Furthermore, the reliability of the test is unclear. Ten experienced surgeons performed hook tests on a cadaver bone model. The applied forces were recorded in a blinded manner. In addition, standardized hook tests with defined forces (50, 80, and 100 N) were performed on 10 pairs of cadaver lower legs and the syndesmosis was sequentially destabilized. Diastasis of the syndesmosis was recorded using an optical 3D camera system. A median force of 81 N (Range: 50 N–145 N) was applied. A proportion of 82% of the tests showed a force < 100 N. The data showed good intraraterreliability and poor interraterreliability. In the standardized investigation of the hook test on the cadaver bone model, both the force and the instability of the syndesmosis had a significant influence on the syndesmosis diastasis. Nevertheless, even with maximum instability of the syndesmosis, diastasis > 2 mm could only be measured in 12 of the 19 evaluable specimens. The widely used hook test shows a high variability when performed in practice. Even in a standardized manner, the hook test cannot detect a relevant syndesmosis injury.

## 1. Introduction

In the surgical treatment of ankle fractures, the question of the integrity of the syndesmosis always arises after stabilization of the bony injuries [1]. The precise detection of any syndesmotic injuries is a basic prerequisite for the correct selection of the treatment strategy and is therefore of great relevance for the long-term outcome [2]. In addition to radiological examination methods like classic X-ray and also CT or MRI [3], arthroscopic procedures or intraoperative direct assessment of syndesmal ligament structures, intraoperative dynamic stress tests [2,4,5,6] for the assessment of stability are used to quantify syndesmosis injuries. A common examination procedure for this purpose is the intraoperative application of the Hook test [7,8,9]. Here, the distal fibula is grasped with a hook and pulled horizontally to the lateral side, while the foot is fixed by the examiner [10]. Subsequently, the extent to which the visible syndesmotic gap is widened is assessed. In addition, the change of the tibiofibular clear space can be monitored using intraoperative X-ray. Another stress test that can be used well intraoperatively is the external rotation stress test, in which an external rotation stress is applied to the foot while the lower leg is fixed. Analogous to the hook test, a widening of the syndesmosis is recorded at the moment the force is applied. In this context, it has also been shown that the assessment of a possible widening of the medial clear space seems to be advantageous [11]. If the medial clear space is used in the intraoperative application of stress tests for the assessment of syndesmal instability, the external rotation stress test seems to be superior to the Hook test [12]. In addition, this test has the uninjured opposite side available for comparison because, unlike the Hook test, this test can be performed noninvasively. Nevertheless, both tests have the disadvantage that inaccuracies can occur in the preparation of the X-ray image at the moment of force application, e.g., if the Mortise view is not acquired in exactly the same rotation as the reference image without stress.

In a comparative biomechanical study published by Stoffel et al. these two intraoperative stability tests were contrasted under laboratory conditions [8]. Using a cadaver model, it was shown that the Hook test (=lateral load test) is better suited to detect syndesmosis instability than the external rotation stress test. Similar results were shown by Jiang et al. [4]. However, justified doubts about the suitability of the Hook test are certainly expressed. For example, the direction of the pulling force seems to play an essential role, whereby the ideal direction in this context seems to lie rather in the sagittal plane [9].

Furthermore, there are no recommendations for the practical performance of the Hook test, regarding the force to be pulled and the optimal direction of force. This is at the surgeon’s discretion. Ingall et al. showed that the applied force when performing the Hook test varies greatly between surgeons [13]. The median (IQR) force applied during the Hook test was 96.42 N (71.42–126.33), and the measured forces were then further differentiated between attendings 87.49 N (69.19–117.40) and trainees 99.99 N (79.91–137.49).

Due to the existing ambiguities from a biomechanical point of view, but also with regard to the lack of recommendations for practical implementation, the present study was designed. First of all, the reliability of the test during practical performance was to be investigated. For this purpose, experienced physicians were involved in a scenario that was as close to reality as possible. Based on the results obtained, a standardized investigation of the hook test in the laboratory using a cadaver model should follow in order to validate this clinical test from a biomechanical point of view.

## 2. Materials and Methods

This study was performed in accordance with the principles of the Declaration of Helsinki. Approval was granted by the Ethics Committee (approval number: 2021-2431-Material).

### 2.1. The Hook Test in Clinical Routine (Experiment A)

**Figure 1:**
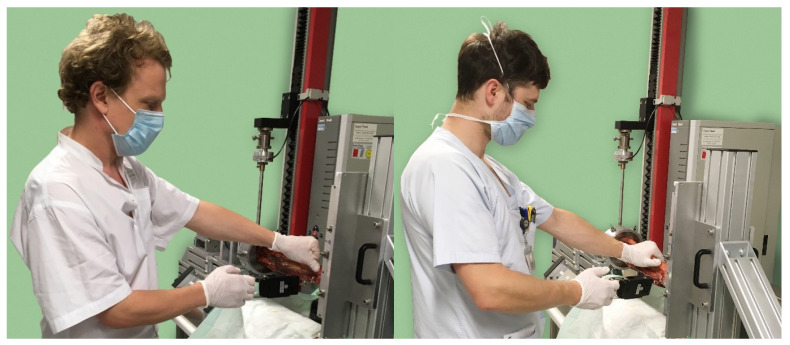
Test setup for experiment A. Two surgeons performing a hook test like they would do intraoperatively. The applied force was measured using a digital portable scale. The surgeons could not see the display of the scale.

To evaluate the individual performance and calculate the reliability of the hook test in clinical practice, a total of 10 experienced surgeons (5× Resident, 2× Specialist, 3× Senior physician) of the local trauma-center conducted the tests. Each one performed a series of five hook tests by applying the force with the right hand that they would use intraoperatively to test syndesmosis stability (See Figure 1). All surgeons were right handed. The applied force was recorded by an independent observer (the surgeons could not see the display of the scale) using an industrial, digital portable scale (measuring range: 0 to 50 kg, accuracy: ±0.001 kg). The hook of the scale was attached to the distal fibula just above the syndesmosis from an intact lower leg specimen. All surgeons used the same lower-leg specimen. The Diastasis was not of interest in this part of the setting; only the surgeon-performed Hook-test-force was in focus, to determine the reliability.

### 2.2. Biomechanical Evaluation of the Standardized Hook Test (Experiment B)

Ten pairs of fresh frozen intact lower legs were used (7 males, 3 females, mean age 87.1 years; range 74–94 years). The specimens were from body donors from the University Anatomical Institute. The specimens were free of accidents or previous operations at the level of the upper ankle joint.

The specimens were thawed over a period of 18 h at room temperature. Subsequently, the soft tissue in the malleolar region was removed, leaving the ligamentous parts of the syndesmosis intact, as well as the deltoid ligament in the region of the medial malleolus and the fibula-calcaneal and fibula-talar ligaments in the region of the lateral malleolus.

**Figure 2:**
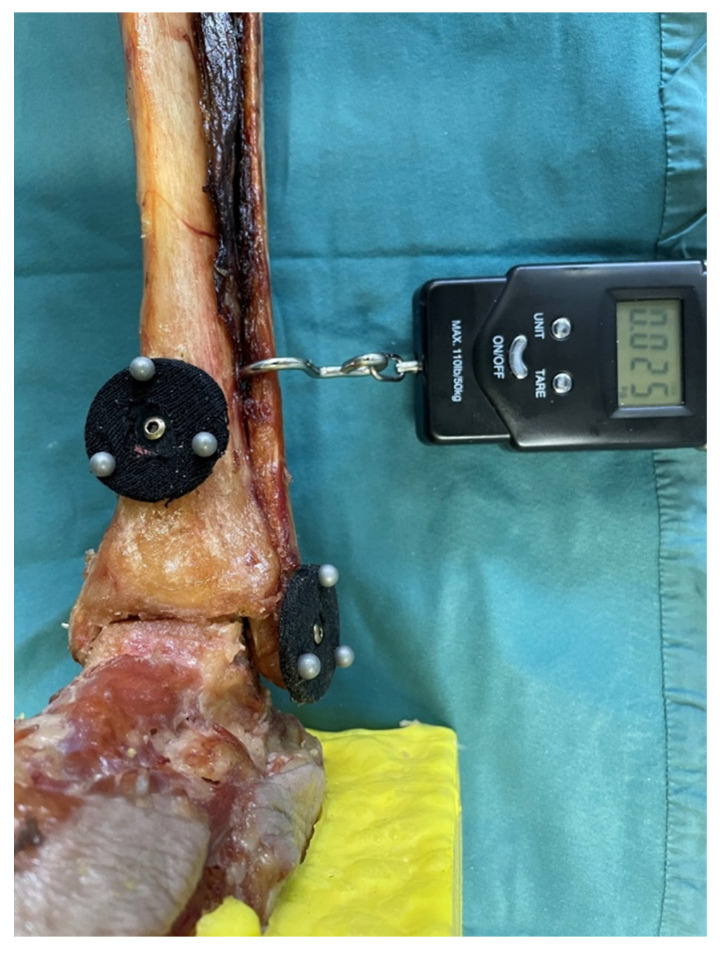
Set-up of experiment B. Prepared lower-leg specimen with attached 3D marker plates and digital portable scale hooked above the syndesmosis.

To detect the movement of the fibula, induced by the force of the Hook test, an optical system was used. Therefore, two passive optical marker plates were used to track the movement with a 3D camera system (3DF scanner with the 3D optical measurement system, kolibri CORDLESS, Germany, resolution 2048 × 1280 pixels, measurement uncertainty 20–100 µm [14]). Both marking plates consisted of a base plate measuring 4 cm in diameter with three reflective marking balls each and were placed in the same manner. The first marker plate was fixed on the distal metaphysis of the tibia 5 cm above the ventral distal edge of the tibia (M1), and the second (M2) on the distal tip of the fibula (see Figure 2). The 3D camera used detected the change in the smallest distance between the two markers. This was, therefore, a combination of movement in the coronal and sagittal planes. A possible rotation of the fibula along the axis was not registered.

To perform the hook tests, the specimens were rigidly fixed proximally in the region of the tibial plateau and distally on the foot. All lower legs, with intact ligaments were initially loaded with the hook test of 50, 80, and 100 N, respectively. To precisely control the applied force, a portable industrial scale was used, which was hooked into the fibula above the intact anterior syndesmosis (see Figure 2). The examiner pulled horizontally laterally and rectangular to the fibula’s axis with the digital scale under permanent control of the applied force. During this process, the change in diastasis, respectively the movement of both markers (M1 and M2) was recorded using the 3D camera system.

To avoid random side effects during subsequent increased instability, the right and the left leg of each pair were randomized in one of two group (A or B).

In group A, the level of stepwise instability of the syndesmosis was performed in the following order (anterior to posterior):

1. transection of the anterior syndesmotic ligament (AITFL);
2. transection of the intermediary syndesmotic ligament;
3. osteotomy of the posterior edge of the tibia (bony release of the PIFTL);
4. transection of the deltoid ligament.

A surgical scalpel was used to cut the ligamentous structures. The osteotomy of the posterior tibial edge was performed with an oscillating saw.

In group B, the level of stepwise instability of the syndesmosis was performed in reverse order of group A, as follows (posterior to anterior):

1. transection of the deltoid ligament;
2. osteotomy of the posterior edge of the tibia (bony release of the PIFTL);
3. transection of the intermediary syndesmotic ligament;
4. transection of the anterior syndesmotic ligament (AITFL).

After each transection step, a hook test with 50, 80 and 100 N was performed and the change in diastasis was detected as described above. As a natural diastasis of 1 mm in the mediolateral direction is known from the literature [15,16,17], a diastasis > 2 mm was defined as the threshold for relevant syndesmosis instability in the present study.

### 2.3. Statistical Methods

For experiment A, the intra-class-correlation coefficient (ICC) for the single measure and absolute agreement was used to calculate the intraraterreliability (intraRR). ICC below 0.50 was considered poor; 0.50 to 0.75, moderate; 0.75 to 0.90, good; and above 0.90, excellent. Fleiss’ Kappa was calculated for the interraterreliability (interRR). The kappa index was interpreted as poor if less than 0.20, fair if 0.20 to 0.40, moderate if 0.40 to 0.60, good if 0.60 to 0.80, and very good if 0.80 to 1.00.

In order to investigate the effect of instability and force on diastasis in experiment B, a general linear model with repeated measures was created for both groups (A and B). The within-subject factors (5 × 3) used were the sequential cutting of the ligaments, from intact to the maximum unstable state (5 levels), and the force levels (3 levels). If the Mauchly’s test for sphericity was significant, the Greenhouse–Geisser *p*-value was used for the main and interaction effects (force and instability). Post hoc pairwise comparisons were carried out with the Bonferroni tests. For both main effects, additional to the *p*-values, the effect sizes (ES) are given as partial eta squared. Values of 0.01, 0.06, or 0.14 indicate small, medium, or large effects, respectively [18].

The frequency of exceeding the threshold of 2 mm diastasis during biomechanical testing was also documented for group A and group B for all strength and instability levels. The threshold for significance was set at *p* = 0.05. The statistical analyses were carried out using SPSS version 27 software. For visual comparison, the results are given as mean and 0.95 confidence interval as error bars. This means that samples with non-overlapping error bars differ significantly, with *p* < 0.05.

## 3. Results

### 3.1. The Hook Test in Clinical Routine (Experiment A)

**Figure 3:**
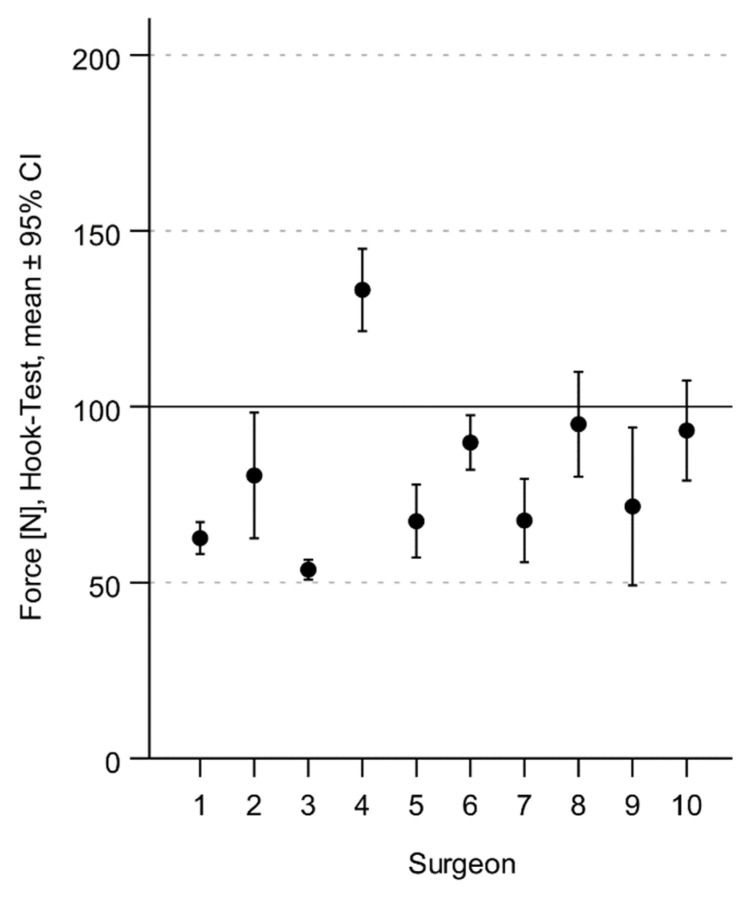
Graphical representation of the forces generated by each of the 10 surgeons.

When the hook test was performed by the surgeons, it was performed at 81 ± 24 N on average. Of the 50 hook tests, 41 (82%) were within the range of 50–100 N. None of the surgeons pulled with a force below 50 N. In nine trials, the surgeons pulled with more than 100 N. The maximum force measured was 145 N. A graphical representation of the forces generated by each surgeon is shown in Figure 3.

The ICC showed with 0.817 (*p* < 0.001) good reliability for intraRR. Nevertheless, significant interRR could not be found (Fleiss’ kappa: −0.024, *p* = 0.188).

### 3.2. Biomechanical Evaluation of the Standardized Hook Test (Experiment B)

Ten lower-leg specimens were used and subjected to the standardized tests described above. One specimen of group B, had a total knee arthroplasty and was excluded. Therefore, group A consisted of *n* = 10 specimens and group B of *n* = 9.

**Figure 4:**
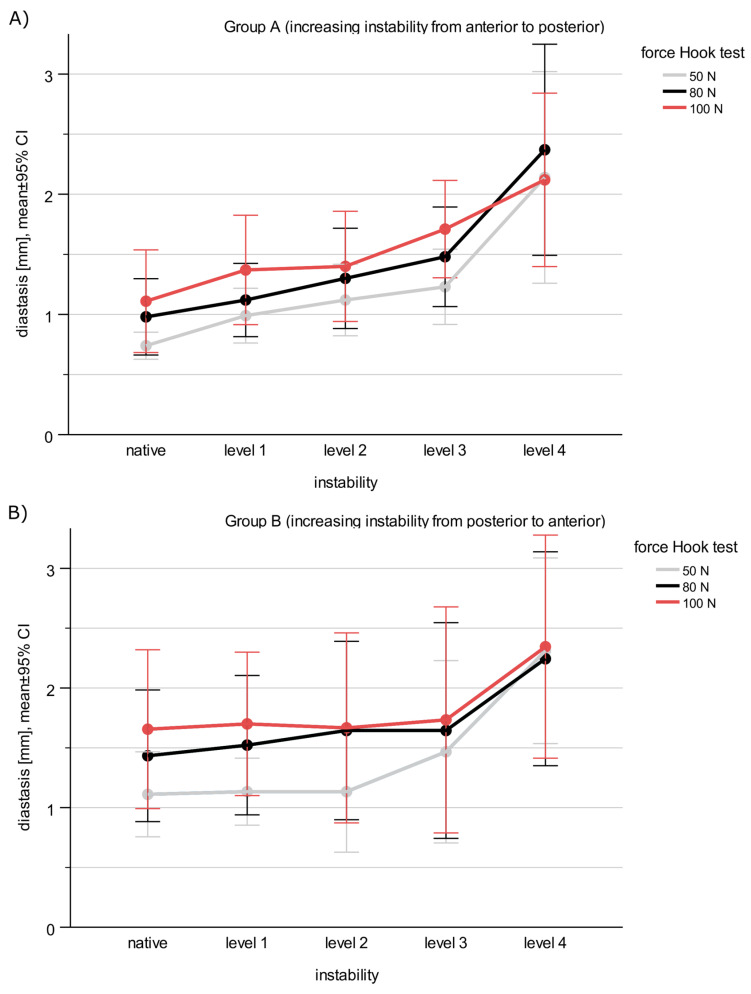
Representation of the diastasis detected in the hook tests in (**A**) group A and (**B**) group B, according to the level of destabilization.

Both the increase of force and the increasing instability of the syndesmosis led to an increase in diastasis in both groups (Table 1 and Figure 4).

Looking at the impact of force on the diastasis, in group A, diastasis increases significantly with increasing force (*p* = 0.003, ES = 0.472) (Table 2). Post hoc pairwise comparisons showed significantly higher diastasis for the hook test at 100 N (*p* = 0.038) and compared to 50 N. Diastasis did not differ between 50 N and 80 N (*p* = 0.058) and between 80 N and 100 N (*p* = 0.362).

Group B also showed a significant increase in diastasis with increasing force (*p* = 0.004, ES = 0.503). Nevertheless, the pairwise post hoc comparisons showed no significant *p*-values for differences in diastasis (50 N vs. 80 N: *p* = 0.057, 50 N vs. 100 N: *p* = 0.046, 80 N vs. 100 N: *p* = 0.372).

The interaction effect of force and instability level showed no significance in both groups (group A: *p* = 0.1081, group B: *p* = 0.179).

With regard to the defined instability threshold of 2 mm, the following results were obtained. In both groups, the measured values of diastasis registered some exceedances of the threshold of 2 mm in the hook test at each degree of instability and at each level of force. Up to 60% (group A) and 67% (group B) of the hook tests showed a diastasis > 2 mm. Table 3 shows the frequency of diastasis above the 2 mm limit depending on the applied force and the degree of instability for each group.

## 4. Discussion

The reliable detection of syndesmosis instability is essential in the management of an ankle injury. Despite the variety of examination methods (preoperative imaging procedures such as sonography or MRI, intraoperative procedures such as ankle arthroscopy or various intraoperative stress tests), all have advantages and disadvantages. No gold standard exists to date. For this reason, the purpose of this study was to investigate whether the hook test is reliable in clinical use and, in addition, whether the test can achieve relevant diastasis at different instability levels under laboratory conditions.

Among the stress tests that can be performed intraoperatively, the hook test and the external rotation stress test are widely used and easy-to-perform methods [12]. In their cadaver study, Stoffel et al. were able to demonstrate the superiority of the hook test, so that this test appears to be superior to the external rotation stress test, at least from a biomechanical point of view [8]. Comparable results were obtained in the cadaver study by Jiang et al. [4].

But does the Hook test really represent the ideal examination method for intraoperative testing of syndesmosis stability?

Candal-Couto et al. were able to show that diastasis of the fibula with an increasing instability of the syndesmosis is more likely to be observed in the sagittal plane, whereas the hook test causes movement in the coronary planes [9]. Similar observations were made by Xenos et al. [15]. Thus, the question of the validity of this test arises, at least when traction is applied in the frontal plane.

Furthermore, there is no recommendation on the strength of force to be applied for a hook test. As a result, a very high variability with regard to the applied force can be observed in the practical performance [13] and it is therefore to be expected that syndesmosis injuries may remain undetected if the force is insufficient or even is misidentified as injured when the native diastasis is over 2 mm.

The aim of the present study was to analyze the suitability of the hook test from two perspectives. First, the force with which the hook test is performed in trauma surgery was investigated. Here, the 10 trauma surgeons provided a very inhomogeneous picture. In addition to a good intraRR, no significant interRR was observed, which is consistent with the observations of Ingall et al. [13] and highlights the extremely variability of the hook test in its practical performance. The majority of the hook tests were performed with a force between 50 and 100 N (82%). From our own observation and the result of Stoffel et al. [8], who showed in their cadaver study no further diastasis of the syndesmosis at a force of more than 100 N, we developed a cadaver model to validate the hook test under standardized conditions in the laboratory. Both the force used and the level of the syndesmosis instability showed a significant effect on diastasis. However, even when using 100 N, the hook test was not able to indicate a diastasis of the syndesmosis of >2 mm in all specimens even if the transection of all relevant ligament structures was performed previously. The results when examining the changes in diastasis at different instability levels show only significant differences in three test setups in both groups. This indicates that the traction applied in this form (horizontal traction to lateral) is not able to detect a relevant syndesmosis injury. In general, however, it must be pointed out that the sample size in the present study was pretty small (Group A: *n* = 10/Group B: *n* = 9). It is possible that the significance/preciseness of the results could be increased if more samples were examined in the manner described. In addition, the specimens came from very old people (mean age 87.1 years; range 74–94 years), so that age-associated diseases, such as osteoporosis, may be present to an over-average extent. Bone densitometry was not performed beforehand. Furthermore, there were more men than women in the specimens used, and, thus, an influence of gender on the significance of the hook test cannot be completely ruled out.

Regarding the force used, we obtained comparable results to Stoffel et al. [8]. An increase of the force from 50 N to 80 N showed a significant influence on the diastasis, while the increase from 80 N to 100 N remained without significant effect.

In our opinion, the following factors are responsible for the poor reliability of the hook test when performing the test in the setup described in the present study.

In the present study, as many as 18 or 29% of the intact specimens already showed a diastasis >2 mm, when the Hook test was performed with 100 N. This indicates a very high individual variability/laxity of the uninjured ankle joint. Relying on the Hook test in practice would therefore mean an overtreatment with an unnecessary syndesmotic stabilization procedure. In contrast, 7 (37%) of the fully instable specimens had a diastasis smaller than 2 mm, which would mean no further stabilization in clinical practice. The very high variability in the practical performance of the Hook test, as shown by the results of this study and the results of Ingall et al. [13], and the possibly non-optimal direction of traction, as reported by Candal-Couto et al. [9], pose further significant problems.

In addition, there is the problem of a constant setting when using X-rays performing a stress test in the operating room, which could be excluded in the present study by using a 3D camera. Even differences of a few degrees in the rotation at the moment of image acquisition can cause a change in the measured radiological parameters (tibio-fibular overlap/clear space, medial clear space) [19] and thus provide an incorrect result.

Further studies should follow in order to improve the applicability of the hook test for detecting syndesmosis instabilities. In particular, with regard to the ideal pulling direction, the appropriate radiological parameter (tibiofibular overlap, medial clear space) and the possibility of intraoperative standardization of the applied pulling force, a modification of the test could definitely increase its informative value.

## 5. Conclusions

In summary, the hook test is a simple intraoperative stress test for checking syndesmosis stability. Due to the very high variability in the practical implementation of the test, the purely monodirectional lateral direction of traction and the high individual variability of the natural syndesmosis flexibility, its results can only be interpreted to a very limited extent.

Therefore, the assessment of syndesmosis stability should not be based on the result of the intraoperative hook test alone.

## Figures and Tables

**Table 1:** Mean values (± standard deviation) of the diastasis (mm) of group A (a: anterior to p: posterior destabilization) and B (posterior to anterior destabilization) for each level of instability, starting in native in the stable condition up to level 4 within the highest level of instability.

Group	Force	Native	Level 1	Level 2	Level 3	Level 4
A (a–p)	50 N	0.7 ± 0.2	1.0 ± 0.3	1.1 ± 0.4	1.2 ± 0.4	2.1 ± 1.2
*n* = 10	80 N	1.0 ± 0.4	1.1 ± 0.4	1.3 ± 0.6	1.5 ± 0.6	2.4 ± 1.2
	100 N	1.1 ± 0.6	1.4 ± 0.6	1.4 ± 0.6	1.7 ± 0.6	2.1 ± 1.0
B (p–a)	50 N	1.1 ± 0.5	1.1 ± 0.4	1.1 ± 0.7	1.5 ± 1.0	2.3 ± 1.0
*n* = 9	80 N	1.4 ± 0.7	1.5 ± 0.8	1.6 ± 1.2	1.6 ± 1.2	2.2 ± 1.2
	100 N	1.7 ± 0.9	1.7 ± 0.8	1.7 ± 1.0	1.7 ± 1.2	2.3 ± 1.2

**Table 2:** *p*-values of the pairwise post hoc comparison of the instability levels for both groups (WITHOUT consideration of the different force levels), a: anterior, p: posterior.

Group		Native	Level 1	Level 2	Level 3	Level 4
A (a–p)	native	\	0.114	0.068	0.012	0.018
	level 1	0.114	\	1.000	0.248	0.053
	level 2	0.068	1.000	\	0.293	0.090
	level 3	0.012	0.248	0.293	\	0.427
	level 4	0.018	0.053	0.090	0.427	\
B (p–a)	native	\	1.000	1.000	1.000	0.105
	level 1	1.000	\	1.000	1.000	0.086
	level 2	1.000	1.000	\	1.000	0.270
	level 3	1.000	1.000	1.000	\	0.031
	level 4	0.105	0.086	0.270	0.031	\

**Table 3:** Frequency of diastasis above the 2 mm limit depending on the applied force and the degree of instability for each group (a: anterior, p: posterior).

Group	Force	Native	Level 1	Level 2	Level 3	Level 4
A (a–p)	50 N	0	0	1	1	5
*n* = 10	80 N	0	1	2	2	6
	100 N	1	1	2	2	4
B (p–a)	50 N	0	0	1	3	6
*n* = 9	80 N	2	2	3	3	4
	100 N	3	2	3	3	5

## Data Availability

The data presented in the study are stored on secure servers at University Hospital Jena. Donor consent forms are stored at the Anatomical Institute of the University Hospital in Jena. The data are available on request from the corresponding author.
